# Bacteria Residing at Root Canals Can Induce Cell Proliferation and Alter the Mechanical Properties of Gingival and Cancer Cells

**DOI:** 10.3390/ijms21217914

**Published:** 2020-10-24

**Authors:** Łukasz Suprewicz, Grażyna Tokajuk, Mateusz Cieśluk, Piotr Deptuła, Teresa Sierpińska, Przemysław Wolak, Tomasz Wollny, Joanna Tokajuk, Stanisław Głuszek, Ewelina Piktel, Robert Bucki

**Affiliations:** 1 Department of Medical Microbiology and Nanobiomedical Engineering, Medical University of Bialystok, Mickiewicza 2c, 15-222 Bialystok, Poland; lukaszsuprewicz@gmail.com (Ł.S.); mticv1@gmail.com (M.C.); piotr.deptula@umb.edu.pl (P.D.); asiatokajuk@gmail.com (J.T.); ewelina.piktel@wp.pl (E.P.); 2 Department of Integrated Dentistry, Medical University of Białystok, M. Skłodowskiej-Curie 24a, 15-276 Bialystok, Poland; grazyna.t1@gmail.com; 3 Dentistry and Medicine Tokajuk, Żelazna 9/7, 15-297 Bialystok, Poland; 4 Department of Prosthetic Dentistry, Medical University of Bialystok, Waszyngtona 13, 15-269 Bialystok, Poland; teresasierpinska@gmail.com; 5 Institute of Medical Science, Collegium Medicum, Jan Kochanowski University of Kielce, IX Wieków Kielc 19A, 25-317 Kielce, Poland; przemyslaw.wolak@ujk.edu.pl (P.W.); sgluszek@wp.pl (S.G.); 6 Holy Cross Cancer Center, Kielce, Stefana Artwińskiego Street 3, 25-734 Kielce, Poland; tomwollny@gmail.com

**Keywords:** oral microbiota, root canal infections, cancer development

## Abstract

Understanding the importance of oral microbiota in human health and disease also leads to an expansion of the knowledge on functional, metabolic, and molecular alterations directly contributing to oral and systemic pathologies. To date, a compelling number of studies have documented the crucial role of some oral cavity-occurring microbes in the initiation and progression of cancers. Although this effect was noted primarily for *Fusobacterium* spp., the potential impact of other oral microbes is also worthy of investigation. In this study, we aimed to assess the effect of *Enterococcus faecalis*, *Actinomyces odontolyticus*, and *Propionibacterium acnes* on the proliferation capability and mechanical features of gingival cells and cell lines derived from lung, breast, and ovarian cancers. For this purpose, we incubated selected cell lines with heat-inactivated bacteria and supernatants collected from biofilms, cultured in both anaerobic and aerobic conditions, in the presence of surgically removed teeth and human saliva. The effect of oral bacteria on cell population growth is variable, with the highest growth-promoting abilities observed for *E. faecalis* in relation to human primary gingival fibroblasts (HGF) and lung cancer A549 cells, and *P. acnes* in relation to breast cancer MCF-7 and ovarian cancer SKOV-3 cells. Notably, this effect seems to depend on a delicate balance between the pro-stimulatory and toxic effects of bacterial-derived products. Regardless of the diverse effect of bacterial products on cellular proliferation capability, we observed significant alterations in stiffness of gingival and lung cancer cells stimulated with *E. faecalis* bacteria and corresponding biofilm supernatants, suggesting a novel molecular mechanism involved in the pathogenesis of diseases in oral cavities and tooth tissues. Accordingly, it is proposed that analysis of cancerogenic features of oral cavity bacteria should be multivariable and should include investigation of potential alterations in cell mechanical properties. These findings corroborate the important role of oral hygiene and root canal treatment to assure the healthy stage of oral microbiota.

## 1. Introduction

The influence of infection within the oral tissues on general health and the implication of oral microbiota for cancer development have recently gained considerable interest among scientists. Some reports strongly encourage the usefulness of monitoring the bacterial composition of salivary microbiota as a biomarker of carcinogenesis and potential pre-malignancy transformations [1,2]. Although oral dysbiosis seems to be of importance in patients diagnosed with gastrointestinal tract cancers [2], its potential role in the development of the pathology in other distant tissues should not be excluded. For this reason, the issue of focal infections resulting from endodontic treatment has recently resurfaced, and calls into question the long-term effect of root canal treatment and root canal microbiota on patient health [3]. To date, a few bacteria, particularly *Streptococcus* sp., *Porphyromonas gingivalis*, *Fusobacterium nucleatum*, and *Actinomyces* sp., have been reported to be implicated in the pathogenesis of oral squamous cell carcinomas and esophageal cancers, in addition to other tumors of the gastrointestinal tract, mainly colorectal and pancreatic cancer [4,5,6,7]. Recent studies have shown that oral-derived bacteria can colonize the intestines, where they persist, and this leads to activation of the intestinal immune system and chronic inflammation via different mechanisms [8].

Odontogenic infections may affect overall health of humans in a variety of ways. To date, three primary mechanisms have been proposed to link such infections to remote organs within the body. The first is a metastatic infection that occurs due to bacteremia, when spreading bacteria are not inhibited by the mononuclear phagocyte system and find an environment in which their growth is promoted. The second is metastatic damage, where bacteria produce exotoxins and proteins that, when secreted from the bacterial organisms, damage the host cells. The last is a metastatic inflammation where bacterial antigens, upon penetrating the bloodstream, react with circulating antibodies to form immune complexes causing acute and chronic inflammatory states in locations where they have accumulated [9,10]. Development of nano-techniques suitable for analysis of single cell physiology makes it possible to expand this group of mechanisms with other possible cancer development inducers. Ever-growing evidence suggest that apart from typical biochemical and genetic disorders occurring when cancer initiates and progress, alterations in nanomechanical features of cells and cell environments should be also taken under consideration.

An increasing number of studies, performed using different cancer cells at varied malignancy stages evidenced a critical role of biomechanical features of the extracellular matrix (ECM) on cancer development and invasion and revealed the crucial impact of alterations in cellular stiffness on cell migration, cellular proliferation, and apoptosis processes [11,12]. In effect, changes in mechanical properties of the single cells and whole tissues were recorded in a number of cancers, including breast, prostate, and bladder [13,14,15]. In one of the studies, Katira et al. demonstrated that changes in the mechanical properties of cancer cells can results in their faster growth when compared to surrounding healthy cells [16]. Molecular analyses revealed that biomechanical changes occurring in the ECM and cellular compartment might activate a spectrum of intracellular signaling pathways, which regulate cellular growth and expression of adhesion molecules [17]. For instance, tissue stiffness was reported to activate the nuclear translocation of the transcription factor TWIST1 in breast cancer cells, resulting in enhanced cell invasion [18]. The above study revealed a novel carcinogenic factor of a purely physical nature and explained how cell cancerous behavior is influenced by biomechanical inducers [16]. For this reason, in recent years, stiffness has become recognized as a highly specific mechanomarker, indicating pathophysiological changes. Nevertheless, no data currently exist on the potential impact of these bacteria and their bacterial-derived products on the mechanical properties of cells, with basic cellular mechanisms being recently presented as one of the major routes for pathogenesis in a variety of cancers [19]. There is also limited knowledge about the possible impact of bacteria, which are recognized as a non-pathogenic component of oral microflora, but are potentially harmful for patients with oral dysbiosis, especially those that are immunocompromised [20]. The majority of odontogenic infections are linked to multiple bacteria, including aerobic, moderately anaerobic, and strictly anaerobic bacteria. Jundt and Gutt conducted a bacteriological study of purulent exudate collected from odontogenic foci, isolating *Streptococcus viridians, Peptostreptococcus*, and *Staphylococcus*, as well as bacteria of *Prevotella*, *Bacteroides*, and *Actinomyces* genera [13]. Knowledge relating to other bacterial species that may access root canals, nevertheless, remains limited.

*Enterococcus faecalis* (*E. faecalis*) is a commensal bacterium of the gastrointestinal tract, strongly associated with root canal infections and detected in up to 77% of teeth with failed endodontic treatment [14,15,21]. Its persistence in such infections is strongly determined by its ability to compete with other microorganisms, invade dentinal tubules, and resist nutritional deprivation [14]. Although it was demonstrated that the prevalence of *E. faecalis* in root canals is associated with the presence of this bacteria in saliva [15], it is currently suggested that *E. faecalis* is not derived from a patient’s own normal microflora but is of exogenous origin [21]. More importantly, *E. faecalis* was suggested to exert a tumorigenic role in the development of colorectal cancer [22,23,24] that might be conditioned by the production of *E. faecalis*-derived extracellular superoxide and hydrogen peroxide, which damages the DNA of colonic epithelial cells [25].

*Actinomyces* spp. is another anaerobic bacterium that plays an important role in root canal infections. Among this group, *Actinomyces israelii*, *A. naeslundii*, and *A. odontolyticus* are commonly isolated in cultures from the root canals [26,27]. Although *A. odontolyticus* is recognized as a non-pathogenic bacterium that is a part of oral cavity microflora, it appears to be involved in infections related to caries, namely exposure of dentinal tubules during cavity preparation and/or leaking restoration [27].

*Propionibacterium acnes* (*P. acnes*) is a Gram-positive anaerobic/aerotolerant opportunistic rod that is part of skin, oral cavity, large intestine, and external ear canal microbiota. *P. acnes* is recognized as a relatively nonpathogenic opportunistic bacteria but is isolated from 2% of root canals with secondary endodontic infections [28,29]. A compelling and ever-growing number of studies has suggested its implication in a wide range of infections and inflammatory conditions, including those related to medical foreign-body implants [29]. Importantly, the presence of *P. acnes* in prostatic tissues of patients diagnosed with prostate cancer was reported to be positively correlated with prostatic inflammation and suggested to be involved in prostate cancer development [30].

In this study, we aim to explore how the presence of opportunistic yet potentially harmful *E. faecalis*, *A. odontolyticus*, and *P. acnes* bacteria and bacterial biofilm-derived products might affect the proliferation and mechanical features of other cells, including both gingival cells and cancer cell lines. We find that although the effect of the tested bacteria on proliferative potential is variable, incubation of gingival and cancer cells with heat-inactivated bacteria and biofilm-derived supernatants significantly alters the cellular stiffness of human primary gingival fibroblasts (HGF) and A549 cells. The analyses suggest that affecting the mechanical properties of cells may be an additional pathophysiological mechanism that results in the development of diseases within oral and distant tissues.

## 2. Results

### 2.1. Stimulation of Gingival and Cancer Cells with Heat-Inactivated Bacteria

**Figure 1:**
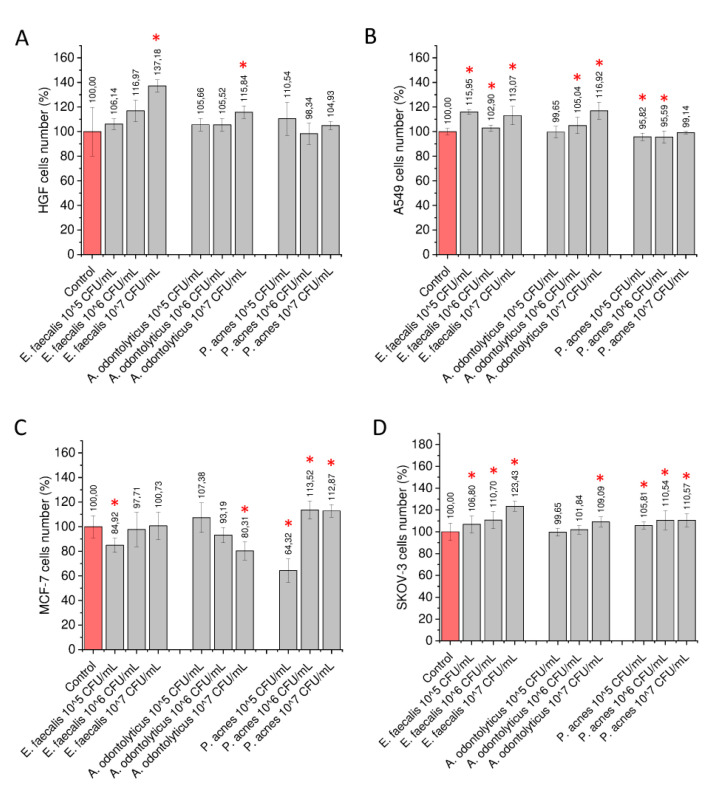
Impact of heat-inactivated bacteria on overall number of gingival and cancer cells. Increase of number of human primary gingival fibroblasts (HGF) (panel **A**), lung carcinoma A549 (panel **B**), breast cancer MCF-7 (panel **C**), and ovarian carcinoma SKOV-3 (panel **D**) cells upon stimulation with *E. faecalis* ATCC 29212, *A. odontolyticus* ATCC 17929, and *P. acnes* ATCC 11827 at concentrations of 10^5^, 10^6^, and 10^7^ CFU/mL. Results are presented as mean ± SD from 6 to 12 individual measurements. * indicates statistical significance (*p* < 0.05) compared to unstimulated control cells.

In the first step of the study, heat-inactivated suspensions of *E. faecalis* ATCC 29212, *A. odontolyticus* ATCC 17929, and *P. acnes* ATCC 11827 were used as an agonist in order to stimulate human gingival cells and lung, breast, and ovarian cancer cells to assess whether their presence might affect the proliferation capability of these cells and, thus, might promote tumorigenesis in oral cavities and in distant tissues. As presented in Figure 1, incubation of both gingival and cancer cells in the presence of a suspension of oral cavity-occurring bacteria, even when physiologically inactive, results in some increase of the number of treated cells.

To characterize the impact of heat-inactivated bacteria on cellular population growth, a fluorometric resazurin-based assay was employed. Resazurin is a well-recognized indicator of cellular viability and proliferation capability, since resazurin-derived fluorescence intensity is correlated with the number of cells and their enzymatic state. As shown in Figure 1, the effect of heat-inactivated bacteria on the proliferation of treated cells is variable and depends on the cancer cell line. In relation to the gingival HGF cell line, a significant increase of cell number was noted when cells were stimulated with *Enterococcus faecalis* ATCC 29212 and *Actinomyces odontolyticus* ATCC 17929 at a bacterial concentration of 10^7^ CFU/mL. When compared to untreated control cells, the ability of gingival cells to proliferate was enhanced by 137.18% ± 5.02% and 115.83% ± 5.13% for *E. faecalis*- and *A. odontolyticus*-treated cells, respectively (Figure 1A). The same tendency was observed in A549 cells, although this cell line was also more prone to exerting stimulatory effects in lower concentrations of bacteria, i.e., ~10^5^ CFU/mL (Figure 1B). Some augmentation of proliferation capability was also recorded for breast cancer MCF-7 cells (Figure 1C) and ovarian cancer SKOV-3 (Figure 1D); notably, a proliferation-promoting effect was also recorded when cells were treated with *P. acnes* ATCC 11827, which was not significant in gingival cells and a lung carcinoma model.

### 2.2. Stimulation of Cells with Biofilm-Collected Supernatants

To investigate whether biofilms of oral cavity bacteria are able to produce and extracellularly release some cancerous factors affecting proliferation capability and viability of surrounding cells, we cultured biofilms of *E. faecalis* in aerobic conditions and *A. odontolyticus* and *P. acnes* biofilms in an anaerobic environment for 24 h, 72 h, and 1 week, and media collected from above the biofilm matrix were used to stimulate gingival HGF cells and lung, breast, and ovarian cancer cells. In a preliminary study, we observed that such prepared samples were highly toxic to both cancerous and non-cancerous cells (data not shown), which is why we decided to dilute the obtained solutions 5- to 25-fold to obtain optimal concentrations of potentially harmful bacterial-derived products.

**Figure 2:**
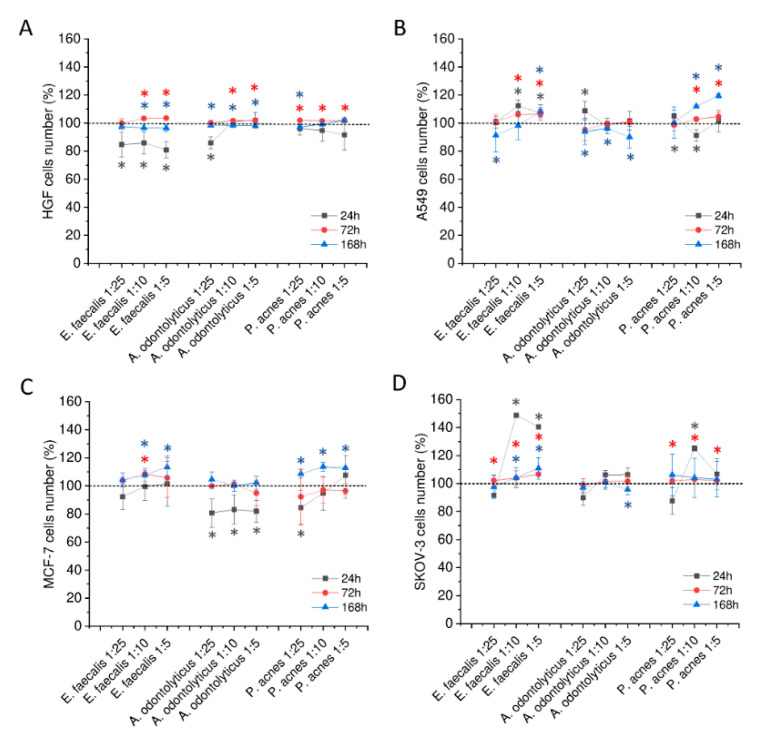
Alterations in doubling potential of gingival and cancer cells upon stimulation with biofilm-collected supernatants. Alterations in number of human primary gingival fibroblasts (HGF) (panel **A**), lung carcinoma A549 (panel **B**), breast cancer MCF-7 (panel **C**), and ovarian carcinoma SKOV-3 (panel **D**) cells upon stimulation with supernatants collected from biofilms of *E. faecalis* ATCC 29212, *A. odontolyticus* ATCC 17929, and *P. acnes* ATCC 11827 in dilutions ranging from 5 to 25. Results are presented as mean ± SD from 6 to 12 individual measurements. Dark grey, red, and blue asterisks indicate statistical significance (*p* < 0.05) when comparing samples incubated for 24, 48, and 72 h, respectively, to unstimulated control cells. Horizontal short dashed line indicates the control, unstimulated cells (100%) to which all results were normalized.

As presented in Figure 2, stimulation of cells with biofilm-derived solutions does not intensely alter the proliferation capability of tested cells, although some increase in cells number after the 168 h treatment was observed, particularly for lung carcinoma A549 cells and breast adenocarcinoma MCF-7 cells subjected to *P. acnes*-collected supernatant (Figure 2B,C). *E. faecalis-*derived solution was also noted to affect the number of SKOV-3 cells (Figure 2D). Those results suggest that the effect of oral cavity bacteria on the proliferation of surrounding cells might be considerably weaker when bacteria are embedded in the biofilm matrix than interacting directly with cells.

Because *Enterococcus faecalis*, a microorganism isolated from the majority of infected root canals, is a facultative anaerobe, we decided to use this bacterium to check the hypothesis that an anaerobic microenvironment is required for bacteria to produce biofilm-derived pro-cancerogenic factors. For this purpose, we cultivated the *E. faecalis* biofilms in both aerobic and anaerobic conditions for 24 and 72 h and diluted collected supernatants 5- to 25-fold in growth medium. The summary of this set of experiments is demonstrated in Supplementary Figure S1. In a majority of combinations, oxygen deprivation does not result in the augmented proliferation of stimulated cells; on the contrary, for MCF-7 cells, a higher growth-promoting effect was noted when *E. faecalis* biofilm was formed in aerobic conditions (Supplementary Figure S1C).

### 2.3. Proliferation Capability of Gingival and Cancer Cells Stimulated with Bacterial Biofilms Formed in the Presence of Tooth Tissue

**Figure 3:**
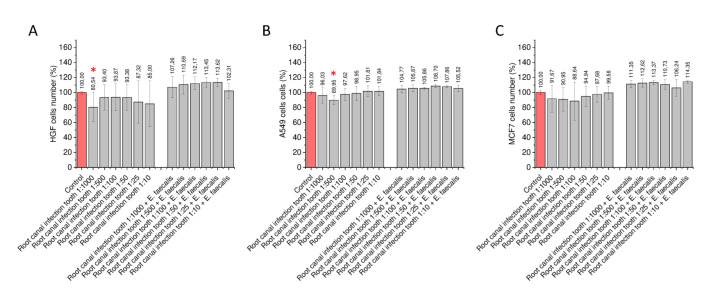
Stimulation of gingival and cancer cells with supernatants of mixed biofilms cultured in the presence of surgically removed tooth in anaerobic conditions and the presence of human saliva. Alterations in proliferation of gingival HGF cells (panel **A**), lung adenocarcinoma cells (panel **B**), and breast cancer MCF-7 cells (panel **C**) upon stimulation with biofilm-derived supernatants. Results are presented as mean ± SD from four individual measurements (*n* = 4). * indicates statistical significance (*p* < 0.05) compared to unstimulated control cells.

To assess the possible impact of biofilm-derived products on the proliferation of gingival and cancer cells, we designed an ex vivo model in which bacterial biofilms were formed (i) in the presence of a tooth, (ii) in anaerobic conditions, and (iii) in the presence of 50% human saliva. Notably, such prepared biofilm-derived supernatants did not exert any stimulatory effect on gingival HGF cells and cancer cells. Some increase in proliferation capability was noted when the culture was co-infected with *E. faecalis*; however, this effect was not statistically significant (Figure 3).

### 2.4. Exposure of Gingival and Cancer Cells to Heat-Activated Bacteria and Biofilm-Collected Supernatants Increase the Number of Ki-67-Positive Cells

**Figure 4:**
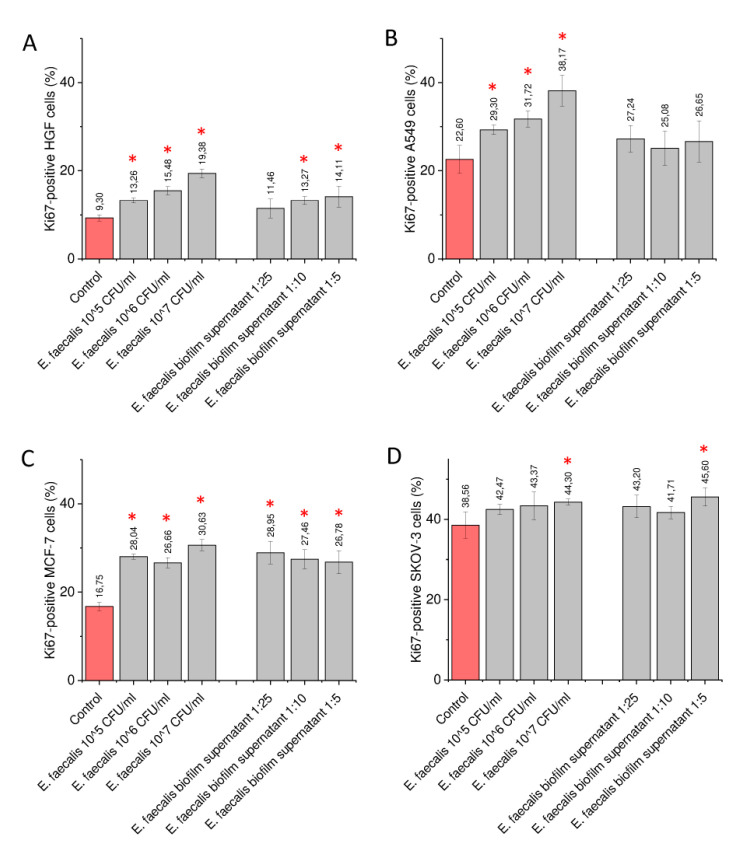
Impact of heat-inactivated *E. faecalis* and bacteria biofilm-derived supernatant on expression of Ki-67 protein in tested cell lines. Alterations in Ki-67 protein expression in human primary gingival fibroblasts (HGF) (panel **A**), lung carcinoma A549 (panel **B**), breast cancer MCF-7 (panel **C**), and ovarian carcinoma SKOV-3 (panel **D**) cells upon stimulation with heat-inactivated *E. faecalis* ATCC 29212 at concentrations of 10^5^, 10^6^, and 10^7^ CFU/mL and biofilm-derived supernatant diluted 5, 10 and 25-fold in culture medium. Results are presented as mean ± SD from 3 individual measurements. * indicates statistical significance (*p* < 0.05) compared to unstimulated control cells.

To confirm the pro-stimulatory effect of heat-inactivated bacteria and biofilm-collected supernatant, we decided to employ an additional test to analyze the proliferation capability of bacteria-treated cells, which is based on measurement of Ki-67 protein level using flow cytometry [31,32]. As is well-recognized, the expression of human Ki-67 protein is associated with cell proliferation exclusively. Since Ki-67 protein is expressed during all active phases of the cell cycle, but not in resting cells (at the G0 stage), Ki-67 expression was established as a sensitive marker for determining the growth fraction of cells [31,32]. As demonstrated in Figure 4, incubation of both gingival and cancerous cells with heat-inactivated bacteria resulted in a statistically significant increase of Ki67-positive cells, which suggests an enhanced proliferation capability within the tested population. This effect was less prominent when bacterial biofilm-derived supernatant was used as an agonist, which is in agreement with our other results.

### 2.5. Morphological and Nanomechanical Features of Treated Gingival and Lung Cancer Cells

**Figure 5:**
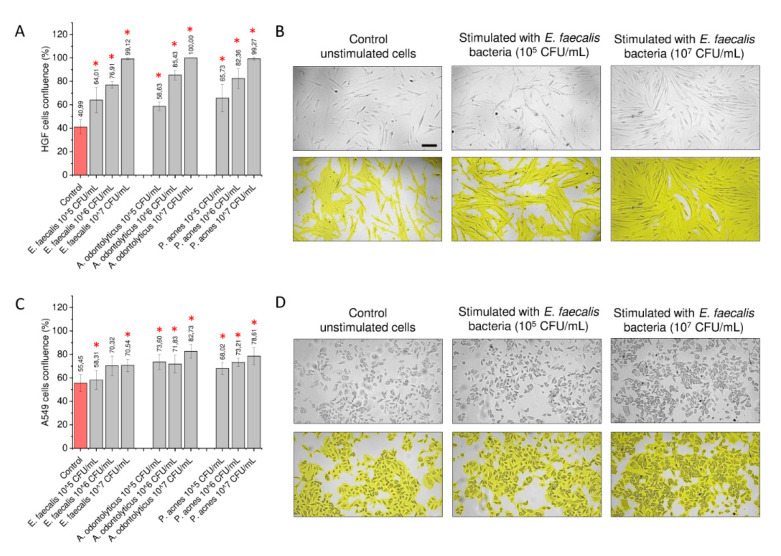
Alterations in morphological features and confluence of gingival and lung cancer cells upon stimulation with heat-inactivated bacteria. Increase of cellular confluence of HGF gingival (panel **A**) and lung carcinoma A549 (panel **C**) cells upon stimulation with heat-inactivated *E. faecalis* ATCC 29212, *A. odontolyticus* ATCC 17929, and *P. acnes* ATCC 11827 for 24 h. Representative microphotographs of HGF (panel **B**) and A549 cells (panel **D**) stimulated with varied concentrations of *E. faecalis* ATCC 29212 for 24 h. * indicates statistical significance (*p* < 0.05) compared to unstimulated control cells. Scale bar ~10 µm.

One parameter that describes the proliferation potential of cells is derived from the measurement of cellular confluence upon incubation with stimulatory factors. Along with the investigation of number of cells by the fluorometric method, we simultaneously evaluated whether cellular confluence is affected by the presence of bacteria and bacteria-derived products, and whether some morphological alterations occur in these cells. Surprisingly, we noted a significant increase in cellular confluence, which suggests a pro-stimulatory effect of oral cavity-occurring microbiota. We also noted that stimulated gingival and lung carcinoma cells became more dispersed on the cell culture surface. No visual indicators of apoptosis or excessive cell death were observed (Figure 5).

**Figure 6:**
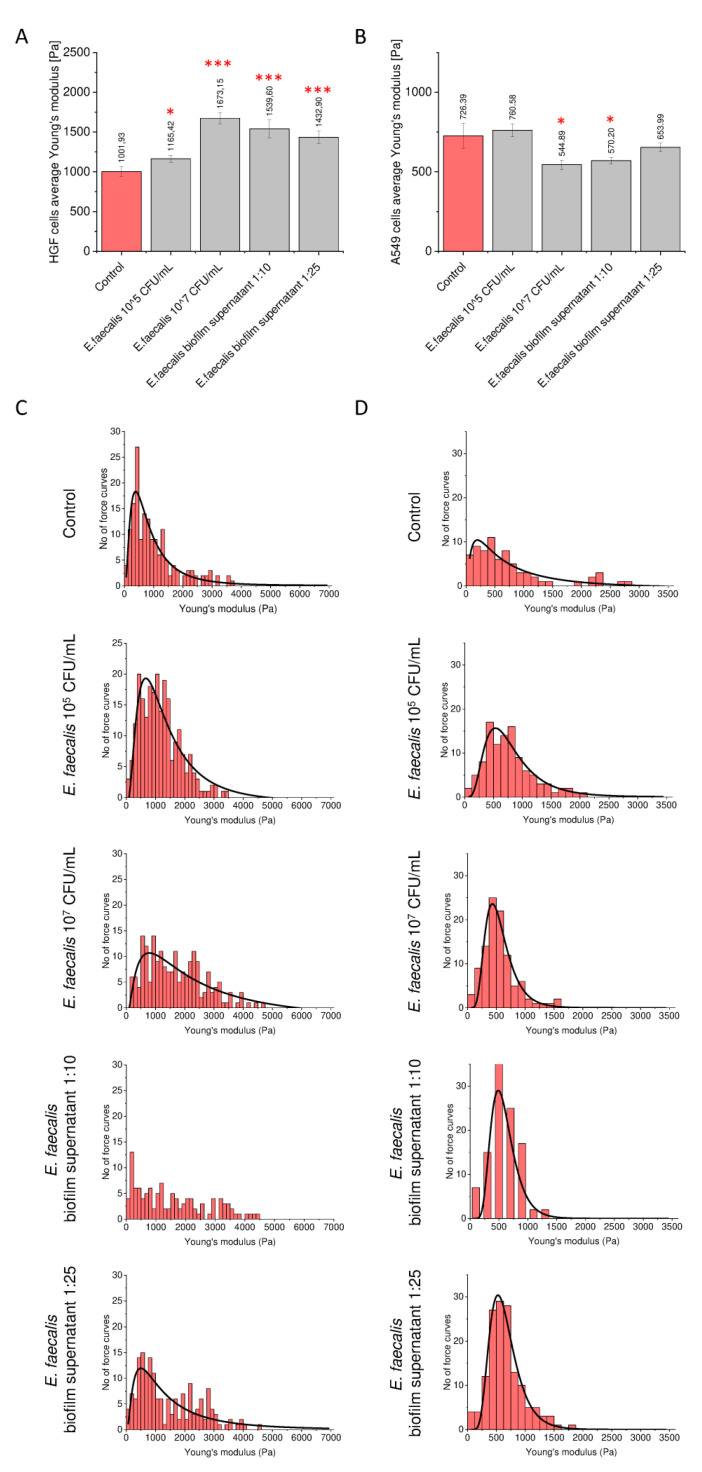
Changes in nanomechanical properties of gingival and lung cancer cells upon stimulation with *E. faecalis* ATCC 29212 determined by AFM using an indentation depth value of 300 nm. Summary of Young’s modulus analysis for HGF (panel **A**) and A549 cells (panel **B**) treated with heat-inactivated *E. faecalis* ATCC 29212 in concentrations of 10^5^ and 10^7^ CFU/mL or biofilm-derived supernatant diluted 10- or 25-fold. Histograms representing the distribution of force curves obtained from tested cells (panels **C**,**D**). Results are presented as mean ± SE. * and *** indicate statistical significance (*p* value < 0.05 and 0.0001, respectively) when compared to unstimulated control cells.

Considering that the morphological features of cells are strongly correlated with the architecture of the cellular cytoskeleton and, thus, with the nanomechanical properties of cells, we hypothesized that some impact of root canal-associated microbes on gingival and cancer cell functions might be connected with changes in the stiffness of these cells. An ever-growing number of studies confirms that alterations in cell mechanical properties induce signaling pathways directly linked with cancer transformation and further malignance. To check this hypothesis, we chose gingival HGF cells and lung carcinoma A549 cells for the analysis of cellular stiffness by atomic force microscopy (AFM) working in force spectroscopy mode using an indentation of 300 µm. As presented in Figure 6, stimulation of cells with heat-inactivated bacteria and bacteria biofilm-derived supernatants resulted in significant changes in Young’s modulus, both in gingival (Figure 6A,B) and cancer cells (Figure 6C,D), although the direction of these changes was opposite. When gingival cells were stimulated, a statistically significant increase in cellular stiffness was recorded, particularly for cells treated with *E. faecalis* bacteria at a concentration of 10^7^ CFU/mL and biofilm-derived solution. More precisely, the average Young’s modulus was 1673.15 ± 69.66 and 1432.89 ± 78.98 Pa for cells stimulated with inactivated bacteria and biofilm-derived supernatant, respectively, whereas control cells were much softer (1001.93 ± 62.57 Pa).

By contrast, stimulation of lung carcinoma A549 cells with *E. faecalis* products directs cells toward having much softer features, resulting in a decrease in the average Young’s modulus from 726.39 ± 78.51 Pa for control cells to 544.88 ± 28.45 Pa for *E. faecalis*-treated cells and 570.19 ± 21.01 Pa in the case of A549 cells stimulated with 10-fold diluted solution collected from bacterial biofilm (Figure 6C,D). The above results indicate that root canal-occurring bacteria significantly affect the mechanical properties of stimulated gingival and cancer cells, which may potentially affect the physiology of these cells.

## 3. Discussion

The identification of new risk factors that may have a significant impact on the development of cancer is critical for effective prevention of this disease and to improve diagnosis. Although public awareness of the harmful effects of improper diet, alcohol abuse, tobacco smoke, and excessive exposure to solar radiation may be considered adequate, knowledge about the implication of oral microbiota in the development of pathological conditions within the oral cavity and distant organs remains low [33]. A number of studies performed to date confirm the important role of oral cavity-occurring microbiota in the pathogenesis of oral, colorectal, and pancreatic cancers, with *Fusobacterium nucleatum* (potential marker for colorectal cancer), *Streptococcus anginosus*, and *Porphyromonas gingivalis* bacteria strongly correlated with oral and head-and-neck region cancers [4,6,34]. Flynn et al. found that the biofilm component of the colonic mucosa of patients with colorectal cancer is consistent with its periodontal biofilm component; for instance, the presence of *Helicobacter pylori* and *P. gingivalis* in the oral microbiota is closely associated with the development of pancreatic cancer. The presence of *P. gingivalis* in the patient’s mouth also suggests a high risk of pancreatic cancer. The oral microbiota of patients with liver cancer also comprises *Clostridium, Oribacterium, Actinomycetes*, and *Campylobacter* bacteria, which currently serve as biomarkers [8]. Zhang et al. concluded that oral squamous cell cancer (OSCC) tissues were enriched in oral bacteria, including *Fusobacterium*, *Alloprevotella*, and *Porphyromonas*, when compared to control tissues, suggesting potential association between these bacteria and OSCC [35]. Most recently, human oral microbiome dysbiosis was also proposed as non-invasive biomarker of colorectal adenoma and colorectal cancer, and a colorectal adenoma group of patients was reported to be characterized by the largest oral microbial composition and diversity [36].

Currently, there is a lack of research investigating the impact of opportunistic oral microbiota on cancer development, as we aim to do in this study. We believe that expanding the knowledge on this issue will be favorable for patients with innate host defense and oral microbiota balance disturbances. It is generally accepted that local infections in the oral cavity, including those occurring in the course of endodontic infections, are mostly driven by disturbances in the local microenvironment of the tooth and the spread of inflammation, which permit progression to the next step of unbalanced host–microbiota interactions [3,37]. The root canal flora of teeth with clinically intact crowns, but having necrotic pulps and diseased periapices, is dominated (>90%) by obligate anaerobes, particularly *Porphyromonas*, *Fusobacterium*, *Prevotella*, *Eubacterium*, and *Peptostreptococcus* [38]. By contrast, the microbial composition, even in the apical third of the root canal of periapically affected teeth, with pulp canals exposed to the oral cavity, not only differs from the root canal flora of teeth with intact crowns but is also less dominated (<70%) by strict anaerobes [38]. Several microorganisms can cause endodontic treatment failure. *Enterococcus* species are prevalent in persistent infections, and *Enterococcus faecalis* has been found to be more common in these infections than other bacteria. Fungi have also been found in primary endodontic infections, but their presence appears to be more prevalent in secondary/persistent endodontic infections and teeth with root canal treatment failure. *Candida albicans* is the most common prevalent fungus in the root canals of infected teeth. Recent studies have also proved the role of viruses such as *Cytomegalovirus* (CMV) and Epstein–Barr virus (EBV) in symptomatic periapical lesions [39,40].

In patients with oral dysbiosis, the consequence of such changes might be an increased virulence of opportunistic pathogens resulting directly from their outgrowth and disrupted effectiveness of the host in responding to them [41]. To date, three mechanisms of actions have been suggested to define the role of oral microbiota in the pathogenesis of cancer: (i) induction of chronic inflammation by bacterial stimulation followed by oncogene activation and mutagenesis, (ii) acceleration of cellular proliferation, affecting cytoskeletal reorganization and inhibition of cellular apoptosis, and (iii) production of some carcinogenic substances, such as reactive oxygen species (ROS), reactive nitrogen species (RNS), and organic acids, which indirectly promote tumorigenesis [34]. Bacteria were also reported to contribute in epithelial to mesenchymal transition [42]. Nevertheless, ever-growing evidence suggests that alterations in nanomechanical features of cells and cell environments should also be taken into consideration [43].

In our study, we aimed to investigate whether the presence of opportunistic oral bacteria might affect viability and nanomechanical features of selected cells lines and to determine the environmental conditions required to achieve the maximum oncogenic effect. For the purpose of this study, we used four cell lines, namely the primary gingival fibroblast cell line derived from adult gingival tissue (HGF) and the three cancer cell lines lung cancer (A549), human breast adenocarcinoma (MCF-7), and ovarian cancer cells (SKOV-3). In the case of the gingival fibroblast line, we decided to use this cell line due to its origin and the fact that its use will reflect the conditions mimicking the oral cavity. The neoplastic lines were selected basis on previous studies indicating the important role of the oral microbiota in the pathogenesis of these neoplasms. Genetic analyses performed to date revealed that the composition of the oral microbiome might determine the development of lung cancer, and certain oral bacteria were found to be potential biomarkers of lung cancer. For instance, Yan X. et al. demonstrated that the amount of *Capnocytophaga* and *Veillonella* are significantly higher in the saliva collected from patients diagnosed with lung cancer [44]. In another study, it was demonstrated that the lung environment has its own microbiome, which influences the occurrence and spreading of lung tumors [45]. Importantly, a compelling number of reports suggest that the lung microbiome originates from the oral microbiome. Additionally, etiological factors of oral and lung infections are similar and often originate from oral microbiota. Although lungs are considered to be sterile, microaspiration of oral fluids is thought to seed the lungs with oral bacteria [46]. In such aspect, some alterations in the lung microbiome were observed as the result of disease states, such as exacerbations in chronic obstructive pulmonary disease [47]. In addition to these reports, large epidemiological studies confirmed that periodontitis is possibly linked to the increase in the relative risk of lung cancer as well [48].

A specific microbial environment was also noted to be associated with the progression of breast cancer [49]. For instance, studies performed by Meurman J. et al. in Sweden suggested that periodontitis should be considered as a breast cancer independent predictor highlighting the role of oral cavity microbial composition [50]. Indeed, women suffering from periodontal disease were found to be more than two times as likely to be diagnosed with breast cancer when compared to healthy subjects [51]. Apart from possible inflammation-associated mechanisms resulting from the periodontal disease and its effect on systemic processes including breast carcinogenesis, the impact of periodontal pathogens should also be taken under consideration. Some studies evidenced the presence of bacteria in breast tissues, including breast tumors [52], and although the exact origin of them has not been established, there are suggestions that they are derived from the oral cavity and gut [53], particularly that some of the bacteria species identified in breast tissues are also found in the mouth [54]. The molecular studies of Wang H. et al. showed that the local breast microbiota differ in patients with and without breast cancer. Increased levels of *Actinomyces* and *Propionibacteriaceae* were spotted in breast tissues, independent of women’s menopausal status [55]. In addition to these reports, use of antibiotics was reported to increase breast cancer risk [56], possibly due to its impact on the oral microbiome, although such a possibility requires further experimental work.

Dysbiosis of the microbiome was also reported to impact the formation and development of ovarian cancer, although the mechanisms underlying this phenomenon are not clear and need to be supported by further experiments. To date, it has been established that composition of the gut microbiome highly disturbs estrogen metabolism, which in turn, promotes gene transcription and mitotic activities of ovarian cancer cells [57]. Considering that the oral cavity and gut share a majority of microbiota species, it cannot be excluded that pathological events in root canals and gingival tissues also affect pathogenesis in distant tissues, including ovaries.

As presented in previous sections, the impact of bacteria, both by direct contact or by stimulation with bacterial-derived products, is variable and cell line-dependent. Although *E. faecalis* ATCC 29212 seems to be “most oncogenic” in our experimental settings (Figure 1), a significant impact of *P. acnes* ATCC 11827 and *A. odontolyticus* ATCC 17929 was also noted, particularly in relation to breast cancer MCF-7 and ovarian cancer SKOV-3 cells (Figure 1). Although some pro-stimulatory impact of anaerobic conditions was also recorded (Supplementary Figure S1), these results were only partially confirmed using an in vitro model of endodontic infection, in which bacterial biofilms were formed in the presence of a surgically removed tooth with opened access to dental pulp (Figure 4). Considering the above, the possible impact of the oral microbiota should be taken into consideration.

Importantly, during the course of our investigation, we also noticed some alterations in morphological (Figure 5) and nanomechanical features (Figure 6) of stimulated gingival and lung cancer cells, suggesting a novel potential oncogenic mechanism based on the rearrangement of cellular architecture, resulting in changes in the stiffness of stimulated cells and leading potentially to pathology. A compelling number of studies demonstrate that endothelial cells or chondrocytes respond to sheer stress and inflammation by increasing their stiffness [58,59]. Importantly, such changes are closely associated with the pathogenesis of osteoarthritis [59], atherosclerosis [60], or vascular inflammation [61]. Simultaneously, higher liver stiffness is associated with a significantly greater risk of hepatocellular carcinoma (HCC) cells [62], and greater stiffness of the microenvironment is considered an independent trigger of epithelial–mesenchymal transition in HCC cells [63]. Interestingly, in malignant tissues, progression of disease and metastasis potential of tumors is augmented when cancer cells decrease their stiffness, as manifested by lower Young’s moduli [64]. Such increased deformability of cancer cells is recognized as a key factor allowing the escape of cancer cells from the primary tumor and subsequent migration [64,65].

Although the aim of our study was not to assess the extent to which bacteria affect the process of cancer cell metastasis and migration, we cannot exclude that such mechanisms exists. Research by Vinay Swaminathan and colleagues performed using a magnetic tweezer system established that as cell phenotypes become more invasive, they display lower cellular stiffness, which results in cell deformation and shape changes favorable for metastasis processes. More quantitatively, cancer cells with the highest migratory and invasive potential were described to be 5-fold less stiff than their non-invasive counterparts [66]. Greater dispersion of lung cancer cells on the cell culture-treated surface might indicate that treated cancer cells are characterized not only by lower cellular stiffness, but also exert enhanced mobility of treated cancer cells and thus greater potential for metastasis. Nevertheless, this issue needs to be thoroughly examined.

The above-cited reports are in great agreement with the data presented in this study. Accordingly, oral bacteria and bacteria biofilm-derived products increase the stiffness of gingival cells, and the resulting transformation is characterized by a more oncogenic phenotype (Figure 6A,B) but, simultaneously, the stiffness of cancer cells decreases, facilitating their metastasis (Figure 6C,D). For this reason, the impact of oral microbes on the nanomechanical balance of surrounding tissues should also be considered. Potentially, such mechanical property-altering impacts of oral microbiota might also contribute to the development of pathogenic conditions in oral cavities and the head-and-neck region. For instance, oral squamous cell carcinoma (OSCC) may be preceded by potentially malignant disorders such as leukoplakia and oral submucous fibrosis (OSF). Oral leukoplakias are often characterized by hyperkeratosis, which is significantly associated with the loss of expression of at least two of the three proteins cornulin, keratin 4, and keratin 13 [67]. The resulting changes in the expression of epithelial cell surface markers may promote colonization of the dysplastic tissue with an altered microbiome enriched with microorganisms such as *Fusobacteria* or *C. albicans*. The levels of colonization on the dysplastic tissue may also be influenced by oral hygiene [68,69].

Recent findings have highlighted the essential roles of oral bacteria in tumor progression and their contribution to tumor formation. Nonetheless, this is just the beginning, and we still lack a systematic framework of the whole process of the effect of oral bacteria on tumor cells and their connections with tumors [70]. In the future, oral microbiota will become a new target for improving the physical state of humans. Our study has some limitations, which should be taken into consideration in future experimental studies. In our experimental setting, we tried to mimic as closely as possible the conditions related to the chronic presence of oral cavity bacteria. Although it would be more favorable to use live bacteria, it should be recognized that the excessive multiplication of bacteria in infected root canals usually forces the dental intervention and thus, application of antibiotics or tooth removal. Live circulating bacteria that enter the blood stream following such activities as tooth brushing or chewing are also rapidly cleared and inactivated by the immune system. For this reason, we decided to evaluate the effect of metabolites, toxins, and components of bacterial cells rather than live bacteria when assessing proliferation and mechanical properties of gingival fibroblasts and neoplastic cells [71].

Such an approach is also justified in the aspect of experiment performing. In cell culture, stimulation of cells with live bacteria is possible only for a limited amount of time, typically not exceeding 6 h. After this time, the number of bacteria significantly exceed the bacteria/cell ratio achievable in the infected environment of the oral cavity or tumor-affected areas, which is determined by the potent capability to actively multiply in high-nutritious growth media. In our experimental settings, we aimed to stimulate gingival and cancerous cells up to 7 days, which is possible only using heat-inactivated bacteria or bacterial biofilm-derived supernatants.

Another limitation is associated with the fact that cellular response to microbial exposition is multivariable and depends on the type of cell line, the specific bacterial or microbial product, as well as its concentration and the duration of the exposure. Since both gingival and cancer cells were subjected to bacteria strains in comparable concentrations and for the same incubation time, we suggest that differences in their effect of tested cell lines is strictly strain- and cell-dependent and is associated with two factors: (1) differences in biochemical structure of bacteria and (2) differences in density and activity of receptors and surface proteins interacting with bacteria. The importance of variations in the chemical structure of components from which bacteria are composed and the meaning of this in the aspect of response of host cells was demonstrated by Hyun Han et al. These authors confirmed that differences in the structure of lipoteichoic acids of *S. aureus* and *S. pneumoniae* bacteria determine the variances in the ability to induce TLR-2 receptors and, thus, to stimulate target cells [72]. Since a similar observation was noted in Gram-negative bacteria [73], it was established that each gram-positive bacterium may have many different inflammatory properties and should be assessed individually. This possibility is additionally supported by the reports demonstrating that the pattern of chemical compounds presented on the surface of cells determine the ability of bacteria to interact with those cells [74,75]. For instance, *Salmonella typhi* was reported to induce proliferation of colon cancer cells when targeting a specific glycosylation pattern, GlcNAcβ1-4GlcNAcβ-N/Gly, on HT-29 cells, while they do not exert any proliferation activities against breast cancer cells MCF-7 due to lack of this pattern [74,75]. Although we do not have confirmation from the literature that oral cavity bacteria exert a similar effect, nevertheless it should be noted that the above tested strains were not tested to date, and obtained data are preliminary only and require further analyses.

Importantly, these data require confirmation using more sensitive molecular methods, particularly in the aspect of possible mechanisms of such activity. It has been established that Gram-positive bacteria are recognized by host cells via interaction with lipoteichoic acid (LTA), soluble extracellular toxins, and membrane peptidoglycans [76]. Although lipopolysaccharide (LPS), an endotoxin of Gram-negative bacteria, is able to solely induce potent shock-associated cellular effects, administration of isolated peptidoglycans and lipoteichoic acid do not always produce an appropriate cellular response, as demonstrated using a bacteria-induced septic shock model [76]. In another study, it was confirmed that cellular response to whole inactivated bacteria and purified peptidoglycans differ significantly [77]. Fiedler et al. demonstrated also that heat-inactivated Gram-positive bacteria, but not isolated LTA, are able to induce proliferation and osteogenic differentiation, which suggests that for the appropriate response of host cells, multifactorial interactions are required [78]. Nevertheless, heat-inactivated bacteria that we used in this study makes it difficult to precisely analyze which compound is responsible for the observed effect. Some assumptions might however be made based on the previous studies. In one of the studies, lipoteichoic acids from Gram-positive *S. aureus* were reported to stimulate proliferation of human non-small-cell lung cancer cells by a mechanism involving TLR-2 activation and release of endogenously formed IL-8, suggesting the crucial role of inflammatory mediators [79]. For the same reason, Gram-positive pneumonia were presented to promote non-small cell lung cancer metastasis [80]. Notably, this mechanism is in agreement with other studies demonstrating a pro-stimulatory effect of pro-inflammatory cytokines [81], which suggest that the effect of tested microbes on both gingival and cancer cells results from an inflammatory response of stimulated cells. The involvement of TLR-2 receptors in the response of cells stimulated with components of Gram-positive cells was also reported by Xie et al., which demonstrated the increase of invasiveness and adhesiveness of breast cancer cells upon bacterial peptidoglycan addition by the NF-ĸB-STAT3-Smad3 signaling pathway [82]. Considering the established link between inflammation state, including those persistent in periodontitis and cancerogenesis [83], we believe that a similar mechanism might be responsible for the effect observed in our research; nevertheless, more detailed analyses are required to confirm such a possibility.

## 4. Materials and Methods

### 4.1. Materials

*Enterococcus faecalis* ATCC 29212, *Actinomyces odontolyticus* ATCC 17929, and *Propionibacterium acnes* ATCC 11827 were obtained from American Type Culture Collection (ATCC, Manassas, VA, USA). Blood agar and Columbia agar with 5% horse blood culture plates were obtained from OXOID (Thermo Fisher Scientific, Waltham, MA, USA). An AnaeroPack system and anaerobic indicators were purchased from Thermo Fisher Scientific (Waltham, MA, USA). Human lung adenocarcinoma cells A549 (ATCC^®^ CCL-185™), human breast cancer cells MCF-7 (ATCC^®^ HTB-22™), human ovarian cancer cells SKOV-3 (ATCC^®^ HTB-77™), and human gingival fibroblasts HGF (ATCC^®^ PCS-201-018™) cells were obtained from ATCC (Manassas, VA, USA). High-glucose Dulbecco’s modified Eagle’s medium (DMEM), McCoy’s 5A cell culture medium, fibroblast basal medium with supplements, and fetal bovine serum (FBS) were ordered from ATCC (Manassas, VA, USA). Trypsin-EDTA, PBS, antibiotic antimycotic 100× solution (10,000 units penicillin, 10 mg streptomycin, and 25 μg amphotericin B per mL), and resazurin sodium salt were obtained from Sigma Aldrich (Saint Louis, MO, USA). Muse™ Ki-67 proliferation kits were purchased from Luminex Corp. (Austin, TX, USA).

### 4.2. Preparation of Bacterial Strains

Bacteria used in this study were cultured in aerobic (*E. faecium* ATCC 29212) or anaerobic conditions (*A. odontolyticus* ATCC 17929, *P. acnes* ATCC 11827). *E. faecalis* ATCC 29212 was cultured on blood agar at 37 °C for 18–24 h before harvesting for experiments. *A. odontolyticus* ATCC 17929 and *P. acnes* ATCC 11827 were grown on Columbia agar supplemented with 5% horse blood at 37 °C in an anaerobic jar using an AnaeroPack™ Anaero anaerobic gas generator and anaerobic indicator for 4 and 7 days, respectively.

### 4.3. Cell Culture

HGF (ATCC^®^ PCS-201-018™), A549 (ATCC^®^ CCL-185™), MCF-7 (ATCC^®^ HTB-22™), and SKOV-3 (ATCC^®^ HTB-77™) cells were cultured according to ATCC-provided user manuals. Briefly, HGF cells were grown in fibroblast basal medium supplemented with rhFGFb (5 ng/mL), L-glutamine (7.5 mM), ascorbic acid (50 µg/mL), hydrocortisone (1 µg/mL), insulin (5 µg/mL), and fetal bovine serum (2%) in the presence of penicillin (50 U/mL) and streptomycin (50 µg/mL). A549 and MCF-7 cells were cultured in high-glucose DMEM supplemented with 10% FBS and 1% penicillin, streptomycin, and amphotericin B. SKOV-3 were grown in ATCC-formulated modified McCoy’s 5A medium supplemented with 10% FBS and 1% penicillin, streptomycin, and amphotericin B. The cells were maintained at 37 °C in an atmosphere containing 5% CO_2_ with saturated humidity. Before experiments, cells were seeded in 96-well clear or black cell culture-treated plates at a density of 5 × 10^3^ cells/well and left for 24 h to adhere and spread.

### 4.4. Evaluation of Proliferation Capability of Treated Cells

At the endpoint of each set of experiments (as described below), the stimulatory medium was removed, and cells were washed twice with PBS in order to remove any residual bacteria or biofilm-derived solutions. In order to assess the proliferation capability of stimulated gingival and cancer cells, a resazurin-based proliferation assay was employed [84]. Briefly, stimulated cells were washed twice with PBS, and resazurin suspended in culture medium was then added to the final concentration of 200 µg/mL. The fluorescence signal was recorded for 60 min using a Varioskan Lux (Thermo Scientific, Waltham, MA, USA) microplate reader using excitation/emission wavelengths of 520/590 nm.

### 4.5. Stimulation of Cells with Heat-Inactivated Bacteria

In a set of experiments assessing the impact of metabolically inactive bacteria on the behavior of other cells, grown bacteria were harvested from culture plates, resuspended in PBS, heat-inactivated by boiling twice for 7 min, and washed with PBS. The efficiency of the heat treatment was confirmed by culturing the bacteria to ensure that there was no growth. Heat-inactivated bacteria were suspended in growth medium to an optical density corresponding to ~10^8^ CFU/mL and diluted serially to adjust the concentration of bacteria to ~10^5^, 10^6^, and 10^7^ CFU/mL. A 100 µL aliquot of prepared bacteria-containing medium was added to gingival cells and cancerous cells on 96-well clear or black plates followed by incubation at 37 °C, 5% CO_2_. Control cells were treated with the same amount of growth medium, but without the bacteria. After 24 h, the medium was removed, the cells thoroughly washed with PBS, and the morphology and overall number of treated cells was evaluated.

### 4.6. Stimulation of Cells with Biofilm-Derived Solutions

To evaluate whether viability and proliferation capability of gingival and cancer cells might be altered by factors produced by bacterial biofilms, the tested cells lines were stimulated with supernatants collected from *E. faecalis* ATCC 29212, *A. odontolyticus* ATCC 17929, and *P. acnes* ATCC 11827 biofilms. In order to do so, bacteria grown on blood agar and Columbia agar with 5% horse blood were harvested, suspended in growth medium to an optical density OD_600_ ~0.1, and cultured for 24 h, 72 h, and 1 week at 37 °C, 5% CO_2_. Then, culture vessels were centrifuged (4500 rpm, 10 min), and supernatant was collected and filtered through 0.22 µm syringe filters (Thermo Fisher Scientific, Waltham, MA, USA) to remove any residual bacteria from the sample. Obtained biofilm-derived solution was plated on growth media to ensure that there was no growth. HGF gingival cells and A549, MCF-7, and SKOV-3 cancer cells were stimulated with this prepared supernatant, diluted in growth medium at 5- to 50-fold, for 24 h and investigated for morphology and overall cell number. The volume of supernatant added to the medium was replaced with the growth medium in the control cells.

### 4.7. Formation of Biofilms in Both Aerobic and Anaerobic Conditions

In order to investigate whether anaerobic conditions might determine the production of some factors with pro-cancerous features, *Enterococcus faecalis* ATCC 29212 biofilms were formed as described in the previous section though simultaneously in aerobic or anaerobic conditions using an AnaeroPack system and anaerobic indicator, and the dilutions in growth medium ranged from 5- to 25-fold. The prepared supernatant was used to stimulate gingival and cancerous cells lines for 24 h before investigating the proliferation capability of treated cells. The volume of supernatant added to the medium was replaced with the growth medium in the control cells.

### 4.8. Stimulation of Cells with Biofilm-Derived Supernatants Formed in the Presence of Bone Tissue

To test the hypothesis of whether cancerous factors might be formed in the presence of both (i) anaerobic conditions and (ii) tooth tissues, an ex vivo model was designed in which bacterial biofilms were formed on an isolated tooth. This study was approved by the Research Ethics Committee of the Medical University of Bialystok (R-I-002/4/2019), and all procedures performed in this study were in accordance with the 1964 Declaration of Helsinki. Exclusion criteria included pregnancy, use of local or systemic antimicrobial agents within 6 months prior to the entry into the study, smoking, diabetes, and other systemic conditions. All samples were collected under strictly aseptic conditions. The teeth were cleaned with 30% hydrogen peroxide (H_2_O_2_) and swabbed with a 2.5% sodium hypochlorite solution (NaOCl). Endodontic access was achieved with a sterile high-speed carbide bur until the root pulp was exposed, and the root canal was irrigated using sterile saline. Whole unstimulated saliva (2 mL) was collected from each patient into sterile plastic containers via the “spitting method”. For saliva samples, patients were not allowed to clean their teeth or to eat 30 min before sampling. They rinsed out their mouths with 10 mL of 0.9% sterile saline for 60 s, and these mouthwashes were collected in sterile plastic recipients. All samples were stored at −80 °C until use.

Teeth were flooded with 5 mL growth medium mixed with filtered human saliva at a 1:1 ratio. In one of the combinations, the sample was additionally infected with *E. faecalis* ATCC 29212 at a concentration of ~10^5^ CFU/mL. Such prepared teeth were each sealed in a 15mL tube, which was then placed into a GasPak jar and transferred to a rotating incubator, where samples were incubated at 37 °C for 72 h (200 rpm) in anaerobic conditions using an AnaeroPack system and anaerobic indicator. To mimic saliva flow in the oral cavity, medium was changed every 24 h without further addition of bacteria. After 72 h, medium above the tooth was collected, filtered through 0.22 µm syringe filters (Thermo Fisher Scientific, Waltham, MA, USA) to remove residual viable bacterial cells, diluted 10- to 1000-fold, and used to stimulate cells for 24 h. At this time point, the proliferation capability was investigated, as described above.

### 4.9. Measurement of Ki-67 Expression

To assess the level of Ki-67 expression, tested gingival and cancer cells were seeded into 12-well clear cell culture plates at density of 5 × 10^4^ cell/well and left for 24 h to adhere. Then, cells were incubated for 24 h with heat-inactivated *E. faecalis* ATCC 29212 and its biofilm derived supernatant. Afterwards, the cells were fixed, permeabilized, stained with an antibody to Human Ki-67-PE, and analyzed (5000 events/sample) using the Muse cell analyzer. Each experiment was performed at least three times.

### 4.10. AFM Analysis

The mechanical properties of HGF cells and lung adenocarcinoma A549 cells were measured by indentation using atomic force microscopy (Nanowizard 4 Bioscience AFM, JPK Instruments, Berlin, Germany). To assess the impact of bacterial material on the stiffness of stimulated cells, HGF and A549 cells were treated with heat-inactivated *E. faecalis* ATCC 29212 bacteria (at concentrations of ~10^5^ and 10^7^ CFU/mL) or *E. faecalis*-derived biofilm supernatant diluted in growth medium 10- and 25-fold for 24 h at 37 °C with 5% CO_2_. Control cells were treated with the same amount of growth medium, but without the bacteria. Immediately before the experiment, cells were placed in a CO_2_-independent buffer (Thermo Fisher Scientific, Waltham, MA, USA) to prevent pH variation of the cellular environment during analysis. Elasticity measurements were performed using AFM working in force spectroscopy mode in liquid conditions and employing cantilevers (ORC8, Bruker, Billerica, MA, USA) with a spring constant of 0.08 N/m. From each tested cell, up to 64 force–indentation curves were collected in a grid of 8 ×  8 pixels corresponding to a scan area of 10  ×  10 µm with a maximal force of 1 nN. For indentation measurements, more than 300 force–distance curves were recorded from at least 10 different cells for each group. To determine the apparent Young’s modulus of different cells, force–indentation curves were fit to the Hertz contact model.

### 4.11. Statistical Analysis

The significance of differences was determined using the two-tailed Student’s *t* test. *p* < 0.05 was considered to be statistically significant. Results are the average of 3–12 individual measurements.

## 5. Conclusions

Based on the above data, it is suggested that the exact characterization of the oncogenic effect of oral microbiota should be defined using multivariable indicators of changes, rather than single parameters in isolation. In addition to measurements of proliferation capability or mutations in stimulated cells, additional parameters, such as cell stiffness, should be taken into consideration. Presently, further research is needed to define the potential carcinogenicity of root canal microbiota.

## Supplementary Materials

The following are available online at https://www.mdpi.com/1422-0067/21/21/7914/s1, Figure S1: Impact of anaerobic conditions on cancerogenic potential of cultured biofilms.

## Abbreviations

*A. odontolyticus* ATCC 17929	*Actinomyces odontolyticus* bacteria strain no. 17929
*E. faecalis* ATCC 29211	*Enterococcus faecalis* bacteria strain no. 29211
*P. acnes* ATCC 11827	*Propionibacterium acnes* bacteria strain no. 11827
A549	human lung adenocarcinoma; cell line isolated from a pulmonary adenocarcinoma
DMEM	Dulbecco’s modified Eagle’s medium
HGF	primary gingival fibroblast cell line derived from adult gingival tissue
MCF-7	human breast adenocarcinoma
PBS	phosphate-buffered saline
SKOV-3	human ovarian adenocarcinoma
