# Supplementary material for: Bacteria Residing at Root Canals Can Induce Cell Proliferation and Alter the Mechanical Properties of Gingival and Cancer Cells
Source: Int J Mol Sci. 2020 Oct 24;21(21):7914. doi: 10.3390/ijms21217914 (PMC7672538; doi:10.3390/ijms21217914)
Supplement: Supplementary file 1 [file ijms-21-07914-s001.zip › 2020 cancer oral microbiota_Supp_Materials.docx]

**Supplementary Figure 1.** **Impact of anaerobic conditions on cancerogenic potential of cultured biofilms.** Alterations in number of human primary gingival fibroblasts (HGF) (panel A), lung carcinoma A549 (panel B), breast cancer MCF-7 (panel C), and ovarian carcinoma SKOV-3 (panel D) upon stimulation with supernatants collected from biofilms of *E. faecalis* ATCC 29212 cultured in both aerobic and anaerobic conditions and diluted 5- to 25-fold. Results are presented as mean ± SD from 6 to 12 individual measurements. Dark grey, red, and blue asterisks indicate statistical significance (*p* < 0.05) when comparing samples incubated for 24, 48, and 72 hours, respectively, to unstimulated control cells. Horizontal short dash line indicate value of control samples (100%) to which all results were normalized.
