# Supplementary material for: Bacteria Residing at Root Canals Can Induce Cell Proliferation and Alter the Mechanical Properties of Gingival and Cancer Cells
Source: Int J Mol Sci. 2020 Oct 24;21(21):7914. doi: 10.3390/ijms21217914 (PMC7672538; doi:10.3390/ijms21217914)
Supplement: Supplementary file 1 [file ijms-21-07914-s001.zip › 2020 cancer oral microbiota_Supp_Materials.pdf]

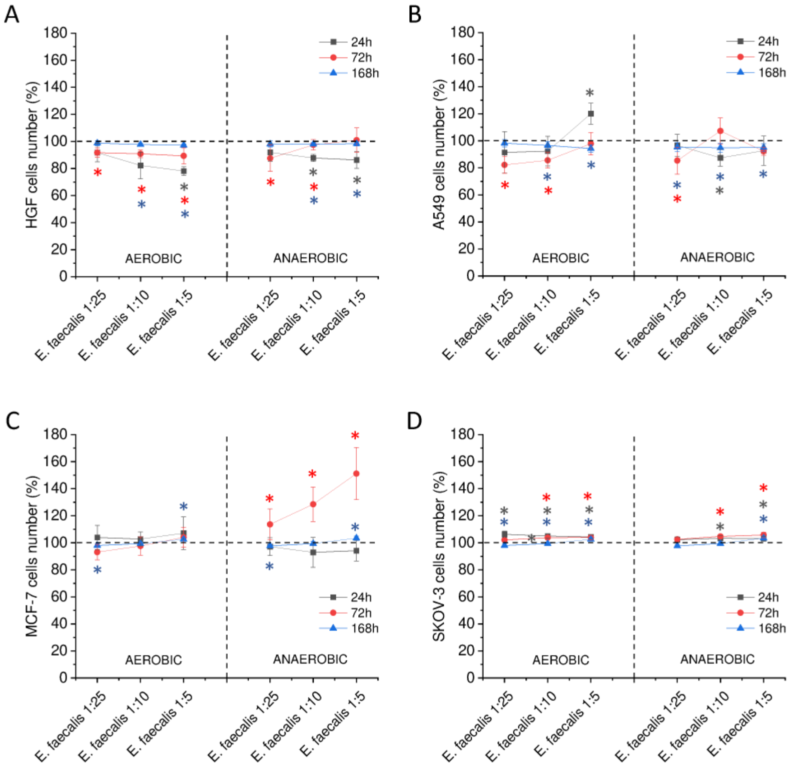

Supplementary Figure 1. Impact of anaerobic conditions on cancerogenic potential of cultured biofilms. Alterations in number of human primary gingival fibroblasts (HGF) (panel A), lung carcinoma A549 (panel B), breast cancer MCF-7 (panel C), and ovarian carcinoma SKOV-3 (panel D) upon stimulation with supernatants collected from biofilms of *E. faecalis* ATCC 29212 cultured in both aerobic and anaerobic conditions and diluted 5- to 25-fold. Results are presented as mean ± SD from 6 to 12 individual measurements. Dark grey, red, and blue asterisks indicate statistical significance (p < 0.05) when comparing samples incubated for 24, 48, and 72 hours, respectively, to unstimulated control cells. Horizontal short dash line indicate value of control samples (100%) to which all results were normalized.
